# Regulation and Function of Laminin A5 during Mouse and Human Decidualization

**DOI:** 10.3390/ijms23010199

**Published:** 2021-12-24

**Authors:** Zhen-Shan Yang, Hai-Yang Pan, Wen-Wen Shi, Si-Ting Chen, Ying Wang, Meng-Yuan Li, Hai-Yi Zhang, Chen Yang, Ai-Xia Liu, Zeng-Ming Yang

**Affiliations:** 1 College of Veterinary Medicine, South China Agricultural University, Guangzhou 510642, China; le1730@126.com (Z.-S.Y.); panhaiyangk@163.com (H.-Y.P.); s18337317812@163.com (W.-W.S.); 15702094622@163.com (S.-T.C.); wangying9816@163.com (Y.W.); 17835422435@163.com (M.-Y.L.); haiyizhang@stu.scau.edu.cn (H.-Y.Z.); yangchen09@yeah.net (C.Y.); 2 Department of Reproductive Endocrinology, Women’s Hospital, School of Medicine, Zhejiang University, Xueshi Road, Hangzhou 310006, China

**Keywords:** decidualization, laminin A5, uterus, PKA, CREB, C/EBPβ

## Abstract

Decidualization is essential to the establishment of pregnancy in rodents and primates. Laminin A5 (encoding by *Laminin α5*) is a member of the laminin family, which is mainly expressed in the basement membranes. Although laminins regulate cellular phenotype maintenance, adhesion, migration, growth, and differentiation, the expression, function, and regulation of laminin A5 during early pregnancy are still unknown. Therefore, we investigated the expression and role of laminin A5 during mouse and human decidualization. Laminin A5 is highly expressed in mouse decidua and artificially induced deciduoma. Laminin A5 is significantly increased under in vitro decidualization. Laminin A5 knockdown significantly inhibits the expression of *Prl8a2*, a marker for mouse decidualization. Progesterone stimulates the expression of laminin A5 in ovariectomized mouse uterus and cultured mouse stromal cells. We also show that progesterone regulates laminin A5 through the PKA-CREB-C/EBPβ pathway. Laminin A5 is also highly expressed in human pregnant decidua and cultured human endometrial stromal cells during in vitro decidualization. Laminin A5 knockdown by siRNA inhibits human in vitro decidualization. Collectively, our study reveals that laminin A5 may play a pivotal role during mouse and human decidualization via the PKA-CREB-C/EBPβ pathway.

## 1. Introduction

Embryo implantation and decidualization are crucial processes for pregnancy establishment in primates and rodents and coordinated by ovarian hormones, growth factors, cytokines, and transcription factors [1]. During decidualization, the fibroblast-like endometrial stromal cells begin to proliferate and differentiate into round epithelial-like decidual cells [2,3]. The decidual tissue promotes uterine remodeling and maternal vasculature development, which are vital for embryonic survival and growth [3,4]. However, the molecular mechanism underlying decidualization is still unclear.

Basement membranes (BMs) are self-assembled and thin sheets of the specialized extracellular matrix and play essential roles in animal growth and development [5,6,7]. BMs are usually found at the interface between connective tissue and parenchyma in peripheral nerve axons, endothelium, muscle cells, and epithelia [8,9]. The major constituents of BMs are fibrous-forming proteins, such as laminin, collagen IV, nidogen, and heparan-sulphate proteoglycans [10,11,12,13]. Minor components include fibulin, agrin, collagen XVIII, and SPARC [14,15,16,17]. Previous studies show that embryo implantation triggers a spatiotemporal expression of BMs [18]. In addition, BMs regulate uterine differentiation during embryo implantation [19,20,21,22].

Laminins are trimeric proteins with α, β, and γ chains [23]. It has been shown that laminins regulate cellular phenotype maintenance, adhesion, migration, growth, and differentiation in vivo and in vitro [24,25]. Mammalian genomes encode five α chains, four β chains, and three γ chains, of which only 16 of the 60 possible laminin isoforms have been confirmed in mammals [26]. The laminin chains show cell- and tissue-specific distribution. The laminin α1 chain is expressed in the embryonic stage and vanishes from the majority of BMs during development to the adult [27]. The heterotrimers containing the α5 chain, particularly laminin α5β1γ1 and α5β2γ1, are the most common isoforms in the adult tissues [27,28]. In mice, laminin α5 null embryos die before embryonic day 17 [29], laminin β2 null embryos develop normally but die between postnatal days 15 and 30 [30], and laminin γ1 deficiency leads to embryonic lethality owing to endoderm differentiation failure [31,32]. A previous study showed that silencing of *Laminin C1* (coded by laminin γ1) impedes uterine decidualization and cytoskeletal remodeling [21]. However, the expression, function, and regulation of laminin A5 (encoding by *Laminin α5*) during early pregnancy are still unknown.

In this study, we found that laminin A5, B2 (coded by laminin β2), and C1 are strongly expressed in mouse decidua. Laminin A5 regulates mouse decidualization through the progesterone-PKA-CREB-C/EBPβ pathway. Laminin A5 is also expressed in human decidua and knockdown of *LAMININ A5* impairs human decidualization.

## 2. Results

### 2.1. Spatiotemporal Expression of Laminin A5, B1, B2, and C1 in the Mouse Uteri during Early Pregnancy

**Figure 1:**
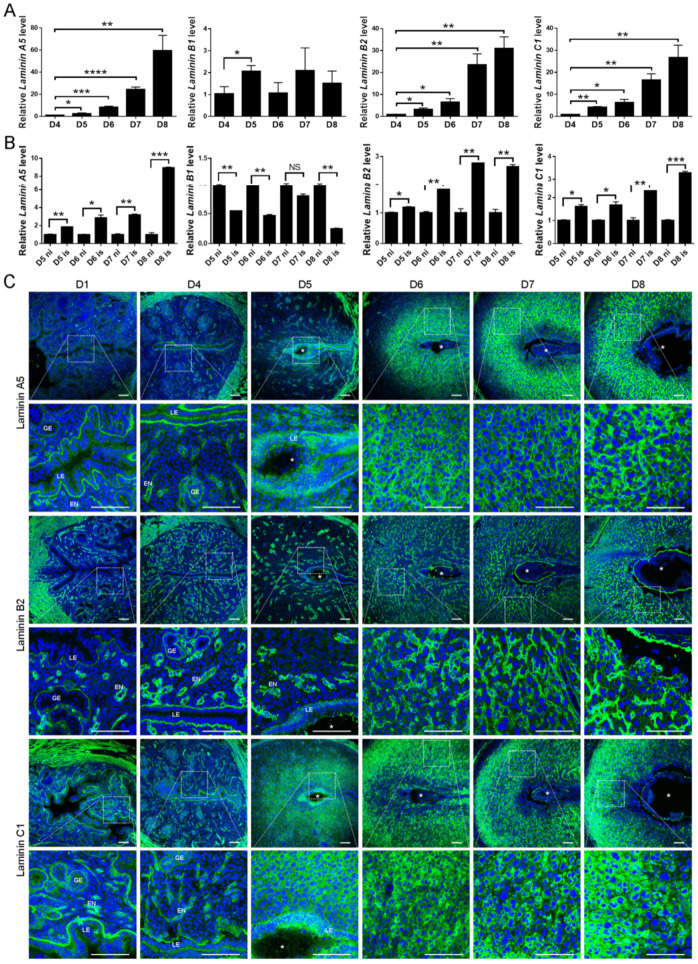
Laminin A5, B1, B2, and C1 expression in mouse uteri during early pregnancy. (**A**) RT-qPCR analysis of *Laminin A5, B1, B2, and C1* mRNA expression from days 4 to 8 of pregnancy. (**B**) RT-qPCR analysis of *Laminin A5, B1, B2, and C1* mRNA levels in mouse uteri at both implantation sites (is) and inter-implantation sites (ni) from days 5 to 8 of pregnancy. (**C**) Immunofluorescence of laminin A5, B2, and C1 proteins from days 1 to 8 of pregnancy. *, embryo; EN, endothelium; GE, glandular epithelium; LE, luminal epithelium. Scale bar = 100μm. Bars represent mean ± SD (* *p*-value < 0.05, ** *p*-value < 0.01, *** *p*-value < 0.001, **** *p*-value < 0.0001). NS, not significant.

Given that heterotrimers α5β1γ1 and α5β2γ1 are the most common isoforms in the adult tissues [27,28], we examined the expression of *Laminin A5*, *B1*, *B2*, and *C1* in mouse uteri during early pregnancy. *Laminin A5*, *B2*, and *C1* mRNA expression was strongly up-regulated from days 4 to 8 of pregnancy (Figure 1A). The mRNA levels of *Laminin A5*, *B2*, and *C1* in implantation sites were significantly higher compared with inter-implantation sites from days 5 to 8 of pregnancy (Figure 1B). However, the mRNA level of *Laminin B1* only increased on days5 (Figure 1A), and significantly down-regulated in implantation sites compared with inter-implantation sites from days 5, 6, and 8 of pregnancy (Figure 1B). To show the tissue localization, immunofluorescence was performed to examine the temporal and spatial distribution of laminin A5, B2, and C1 in mouse uteri. During the preimplantation period (days 1 and 4), laminin A5, B2, and C1 were mainly localized in the basement membrane of the uterine epithelium and endothelium (Figure 1C). Laminin A5 signals disappeared at the basement membrane underlying luminal epithelium at implantation sites on day 5 of pregnancy might be evidence that the embryo will be implanted through epithelial cells (Figure 1C). Similar disappearance of both laminin B2 and C1 was also observed at implantation sites (Figure 1C). Laminin A5 and B2 staining was increased in decidua on days 6 to 8 of pregnancy, whereas laminin C1 was strongly up-regulated in decidua from days 5 to 8 (Figure 1C). These results suggested that heterotrimer α5β2γ1 is the dominant isoform during decidualization.

**Figure 2:**
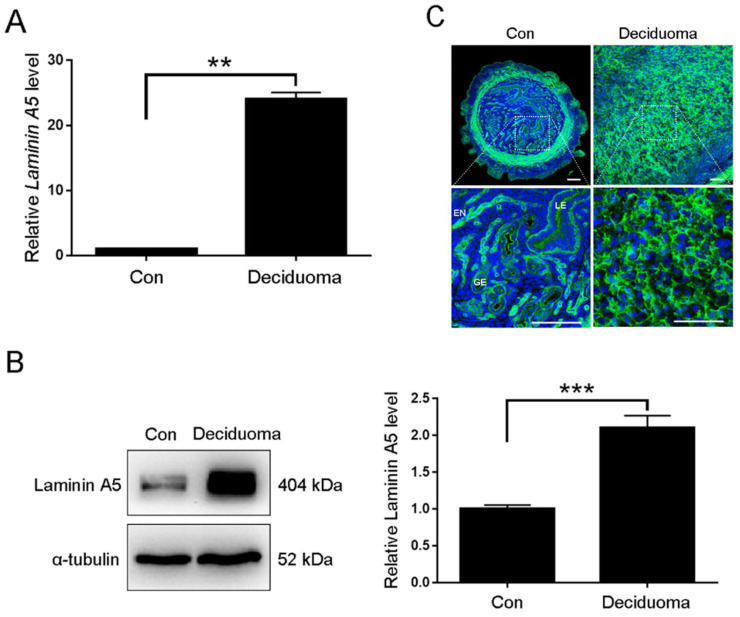
Laminin A5 expression during artificial decidualization. (**A**) RT-qPCR analysis of *Laminin A5* mRNA level. (**B**) Western blot analysis of laminin A5 protein level. (**C**) Laminin A5 immunofluorescence. EN, endothelium; GE, glandular epithelium; LE, luminal epithelium. Scale bar = 100μm. Bars represent mean ± SD (** *p*-value < 0.01, *** *p*-value < 0.001).

Among laminin α5, β2, and γ1, the regulation and role of laminin C1 during mouse decidualization was previously reported [21]. Because laminin A5 was strongly expressed in decidual cells from days 6 to 8, we chose laminin A5 for further analysis. The artificial decidualization model was used to examine whether laminin A5 expression was dependent on embryos. Both laminin A5 protein and mRNA levels were significantly increased in deciduoma (Figure 2A,B). Compared to the uninjected uterine horn, laminin A5 was strongly expressed in decidual cells at the oil-induced uterine horn (Figure 2C).

### 2.2. Effects of Laminin A5 on the Mouse In Vitro Decidualization

**Figure 3:**
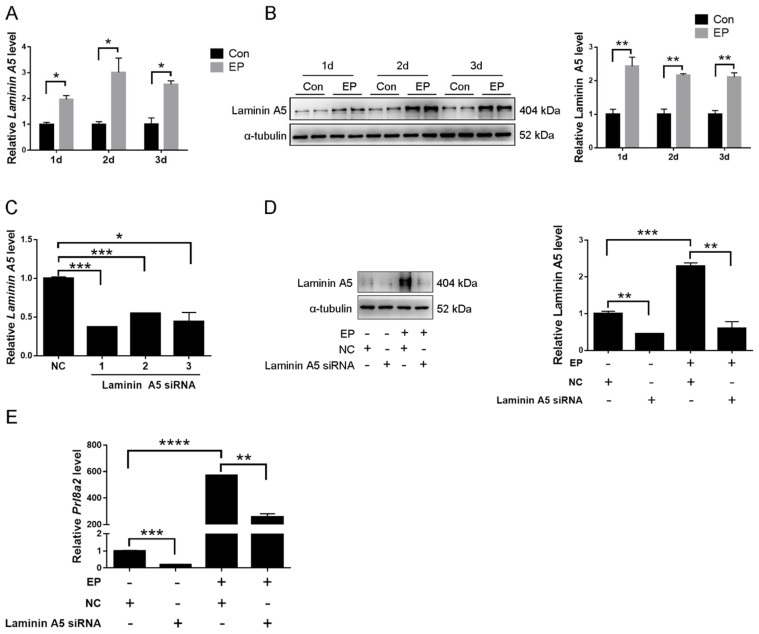
Regulation and function of laminin A5 during mouse in vitro decidualization. (**A**) RT-qPCR analysis of *Laminin A5* mRNA level. (**B**) Western blot analysis of laminin A5 protein level. (**C**) RT-qPCR analysis on the effect of *Laminin A5* siRNAs on *Laminin A5* mRNA level in stromal cells. (**D**) Western blot analysis of laminin A5 protein level after decidualized stromal cells were treated with *Laminin A5* siRNA. (**E**) Effects of *Laminin A5* siRNA on *Prl8a2* expression. Bars represent mean ± SD (* *p*-value < 0.05, ** *p*-value < 0.01, *** *p*-value < 0.001, **** *p*-value < 0.0001). EP, 17β-estradiol + progesterone; NC, negative control.

To examine whether laminin A5 was involved in mouse decidualization, in vitro decidualization was performed. Under in vitro decidualization, laminin A5 mRNA and protein levels were significantly induced (Figure 3A,B). To investigate the role of laminin A5 during decidualization, the efficiency of *Laminin A5* siRNA was examined. Although all three siRNA sequences could remarkably down-regulate *Laminin A5* mRNA levels compared with negative control, siLaminin A5-1 showed the most inhibitory effect (Figure 3C). Thus, siLaminin A5-1 was chosen for further analysis. Prolactin family 8, subfamily A, member 2 (Prl8a2) is a reliable marker for in vitro decidualization in mice [33]. Compared to control, laminin A5 and *Prl8a2* expression were significantly suppressed by *Laminin A5* siRNA under in vitro decidualization (Figure 3D,E). These results suggested that laminin A5 might play a key role during mouse decidualization.

### 2.3. Laminin A5 Regulation by Progesterone

**Figure 4:**
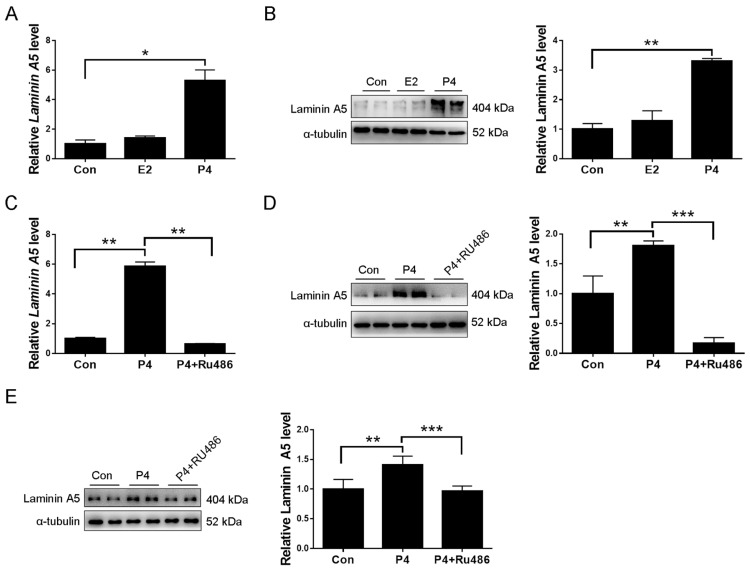
Progesterone regulation of laminin A5 in uterine stromal cells. (**A**) RT-qPCR analysis of *Laminin A5* levels after stromal cells were treated with 1 mM progesterone or 10 nM 17β-estradiol for 72 h. (**B**) Western blot analysis of laminin A5 protein levels after stromal cells were treated with progesterone or 17β-estradiol for 72 h. (**C**) Effects of RU486 on progesterone-stimulated *Laminin A5* expression. (**D**) Western blot analysis on the effect of RU486 on progesterone-stimulated laminin A5 protein level. (**E**) Western blot analysis of effects of RU486 on progesterone induction on laminin A5 expression in ovariectomized mouse uteri. Bars represent mean ± SD (* *p*-value < 0.05, ** *p*-value < 0.01, *** *p*-value < 0.001). P4, progesterone; E2, 17β-estradiol.

Progesterone and estrogen are essential for mouse and human decidualization [34,35]. When uterine stromal cells were treated with 17-β-estradiol or progesterone, 17β-estradiol had no detectable effects on *Laminin A5* level, while progesterone significantly stimulated *Laminin A5* expression (Figure 4A). Western blot also showed that laminin A5 protein level was increased by progesterone (Figure 4B). Progesterone induction of laminin A5 expression was blocked by RU486, a progesterone receptor antagonist (Figure 4C,D). In addition, ovariectomized mice were used to examine whether progesterone regulates laminin A5 expression in vivo. Western blot showed that progesterone significantly increased laminin A5 expression, which was abrogated by RU486 (Figure 4E). These results suggested that progesterone could stimulate laminin A5 expression.

### 2.4. Progesterone Regulates Laminin A5 Expression through the cAMP-PKA Pathway

**Figure 5:**
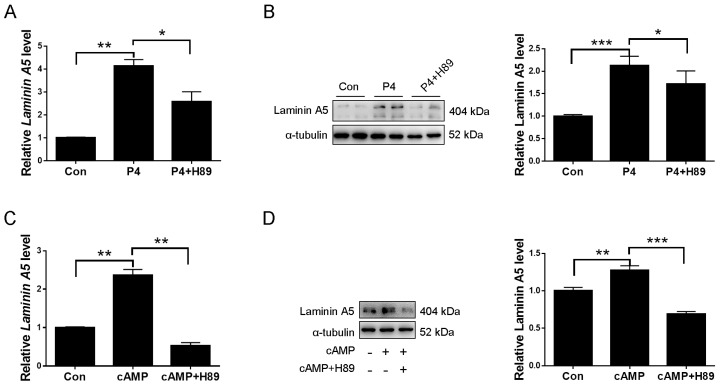
Progesterone regulates laminin A5 through the cAMP-PKA signal. (**A**) RT-qPCR analysis of effects of PKA inhibitor H89 on progesterone-induced *Laminin A5* expression. (**B**) Western blot analysis of effects of H89 on progesterone-induced laminin A5 expression in stromal cells. (**C**) RT-qPCR analysis on effects of H89 on cAMP-induced *Laminin A5* expression after stromal cells were treated with dB-cAMP. (**D**) Western blot analysis of effects of H89 on cAMP-induced laminin A5 expression. Bars represent mean ± SD (* *p*-value < 0.05, ** *p*-value < 0.01, *** *p*-value < 0.001).

cAMP is a critical player during human decidualization primarily via the cAMP-protein kinase A (PKA) signaling pathway [36]. Progesterone can promote intracellular cAMP levels in uterine stromal cells [37]. Therefore, we examined whether cAMP-PKA is involved in progesterone regulation on laminin A5. Progesterone induction on laminin A5 expression was abrogated by PKA inhibitor H89 (Figure 5A,B). Both laminin A5 mRNA and protein levels were significantly up-regulated by cAMP analog dibutyryl cAMP (db-cAMP), which was suppressed by PKA inhibitor H89 (Figure 5C,D). Our data suggested that the cAMP-PKA signaling pathway mediates progesterone stimulation on laminin A5.

### 2.5. CREB-C/EBPβ Mediates the Stimulation of Progesterone on Laminin A5 Expression

**Figure 6:**
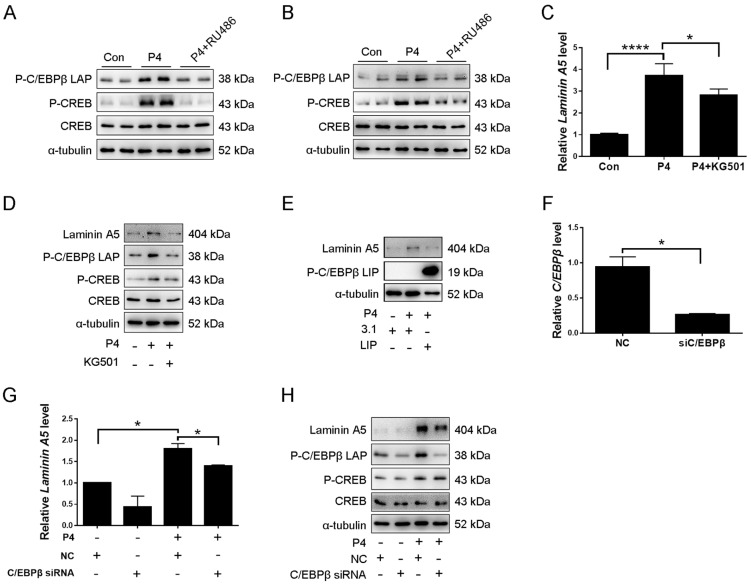
CREB-C/EBPβ mediates progesterone induction on laminin A5 expression. (**A**) Western blot analysis of effects of RU486 on progesterone induction on p-CREB and p-C/EBPβ-LAP expression in ovariectomized mouse uteri. (**B**) Western blot analysis of effects of RU486 on progesterone induction on p-CREB and p-C/EBPβ-LAP expression in cultured stromal cells. (**C**) RT-qPCR analysis on effects of CREB inhibitor KG-501 on progesterone induction of *Laminin A5* expression in stromal cells. (**D**) Western blot analysis on effects of KG-501 on progesterone induction of laminin A5, p-CREB, and p-C/EBPβ-LAP expression. (**E**) Western blot analysis on the effect of C/EBPβ-LIP overexpression on progesterone induction of laminin A5 expression. (**F**) RT-qPCR analysis of *C/EBPβ* mRNA levels after stromal cells were treated with *C/EBPβ* siRNA. (**G**) RT-qPCR analysis of effects of *C/EBPβ* siRNA on progesterone induction of *Laminin A5* expression. (**H**) Western blot analysis of effects of *C/EBPβ* siRNA on progesterone induction of laminin A5, p-CREB, p-C/EBPβ-LAP expression. Bars represent mean ± SD (* *p*-value < 0.05, **** *p*-value < 0.0001). 3.1, empty pcDNA 3.1 (+) vector; LIP, C/EBPβ-LIP overexpression plasmid.

Transcription factor CCAAT enhancer-binding protein β (C/EBPβ) is critical for mouse decidualization in mice and directly controls the *Laminin C1* gene [21]. Furthermore, it has been shown that cAMP induction of *Ass1* and *Dio3* transcription through the PKA-CREB (cAMP-response element-binding protein) pathway [38,39]. In addition, in activated primary macrophage, CREB mediated induction of C/EBPβ expression [40]. Therefore, we were wondering whether CREB-C/EBPβ signaling is involved in regulating laminin A5. When ovariectomized mice were treated with progesterone, the protein levels of p-C/EBPβ LAP and p-CREB expression were significantly increased, which were abrogated by RU486 (Figure 6A and Figure S1A,B). The protein levels of p-C/EBPβ LAP and p-CREB were also induced by progesterone treatment in cultured mice stromal cells, which were significantly suppressed by RU486 (Figure 6B and Figure S1C,D). In cultured stromal cells, progesterone induction on the levels of laminin A5, p-C/EBPβ LAP, and p-CREB were abrogated by KG-501, an inhibitor of CREB (Figure 6C,D and Figure S1E–G). To further investigate whether C/EBPβ is involved in progesterone induction on laminin A5 expression, stromal cells were transfected with C/EBPβ-LIP overexpression plasmid, which is inhibitory form of C/EBPβ. Progesterone induction on laminin A5 expression was evidently reduced by C/EBPβ-LIP (Figure 6E and Figure S1H). *C/EBPβ* siRNA was also used to confirm this regulation. RT-qPCR showed that *C/EBPβ* siRNA significantly down-regulated *C/EBPβ* mRNA level (Figure 6F). Progesterone stimulation on laminin A5 and p-C/EBPβ LAP expression was suppressed by *C/EBPβ* siRNA (Figure 6G,H and Figure S1J,K). However, *C/EBPβ* siRNA had no effect on progesterone up-regulation of p-CREB (Figure 6H and Figure S1L). Our results indicated that progesterone should promote laminin A5 expression through the CREB-C/EBPβ pathway.

**Figure 7:**
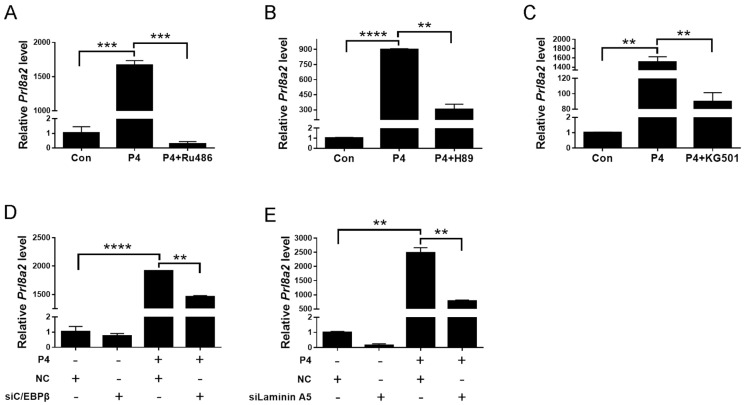
PKA-CREB-C/EBPβ-laminin A5 pathway mediated the effects of progesterone on decidualization. (**A**) Effects of RU486 on progesterone induction of *Prl8a2* mRNA level. (**B**) Effects of H89 on progesterone induction of *Prl8a2* expression. (**C**) Effects of KG501 on progesterone induction of *Prl8a2* expression. (**D**) Effect of *C/EBPβ* siRNA on progesterone induction of *Prl8a2* mRNA level. (**E**) Effect of *Laminin A5* siRNA on progesterone induction of *Prl8a2* mRNA level. Bars represent mean ± SD (** *p*-value < 0.01, *** *p*-value < 0.001, **** *p*-value < 0.0001).

We next determined whether progesterone-PKA-CREB-C/EBPβ-laminin A5 is involved in mouse decidualization. When stromal cells were treated with progesterone, the expression of *Prl8a2* was significantly induced, which was abrogated by RU486 (Figure 7A). In addition, progesterone induction on *Prl8a2* expression was remarkably reduced by H89, KG501, *C/EBPβ* siRNA, and *Laminin A5* siRNA, respectively (Figure 7B–E). Our results indicated that laminin A5 should mediate the regulation of the progesterone-PKA-CREB-C/EBPβ on mouse decidualization.

### 2.6. Expression and Function of Laminin A5 during Human Decidualization

**Figure 8:**
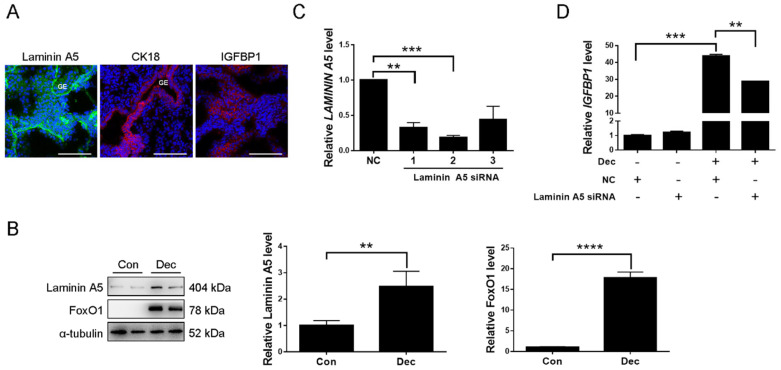
Expression and function of laminin A5 during human decidualization. (**A**) Immunofluorescence of laminin A5, CK18, and IGFBP1 expression in the human first-trimester decidua. GE, glandular epithelium. Scale bar = 100 μm. (**B**) Western blot analysis of laminin A5 and FoxO1 protein levels after human stromal cells were induced for in vitro decidualized with MPA and db-cAMP for 6 days. (**C**) RT-qPCR analysis of *LAMININ A5* mRNA level after human stromal cells were treated with *LAMININ A5* siRNAs. (**D**) Effects of *LAMININ A5* siRNA on *IGFBP1* mRNA level under in vitro decidualization. Bars represent mean ± SD (** *p*-value < 0.01, *** *p*-value < 0.001, **** *p*-value < 0.0001). Dec, db-cAMP + MPA.

Immunofluorescence was used to investigate the expression of laminin A5 in human first-trimester decidua. Laminin A5 signals were detected in decidual cells and basement membranes of uterine glands BMs, in which uterine glands were confirmed by CK18 (Figure 8A). Insulin growth factor binding protein 1 (IGFBP1), a reliable marker for human decidualization [41], was also detected in decidual cells (Figure 8A). When human stromal cells were induced for decidualization, the level of LAMININ A5 protein was significantly increased. The transcription factor Forkhead Box O1 (FoxO1), a reliable marker for human in vitro decidualization [42], was also obviously increased under human in vitro decidualization (Figure 8B). To investigate whether laminin A5 participates in regulating human decidualization, *LAMININ A5* siRNA was used. RT-qPCR showed that the No. 2 fragment of Laminin A5 siRNAs was the most effective sequence for inhibiting *LAMININ A5* expression (Figure 8C). Under in vitro decidualization, the *IGFBP1* mRNA level was significantly repressed by Laminin A5 siRNA transfection (Figure 8D). Our data suggested that LAMININ A5 may be involved in human decidualization.

## 3. Discussion

Our study first identified that laminin A5, B2, and C1 are strongly expressed in mouse decidua from days 5 to 8 of pregnancy, indicating that laminin α5β2γ1 may be involved in mouse decidualization. Although a previous study showed that silencing of *Laminin C1* impedes mouse decidualization [21], the expression, regulation, and function of laminins A5 and B2 during mouse decidualization remains undefined. In this study, we showed that laminin A5 mediates progesterone regulation of decidualization through PKA-CREB-C/EBPβ pathway.

In the tumor, the tumor cells must penetrate the basement membrane during metastasis and invasion. Our results showed that the disappearance of laminin A5 from the subepithelial basement membrane at the implantation site might be evidence that the embryo will be implanted through invading basement membrane. Signal transducer and activator of transcription 3 (STAT3), a marker of mouse uterine receptivity, is crucial for embryo implantation and decidualization [43]. Laminin α5β2γ1 enhances colorectal cancer cell self-renewal through STAT3 activation [44]. The results suggest that laminin α5β2γ1 may regulate the STAT3 activation during decidualization. Mesenchymal-epithelial transition (MET) is involved in decidualization, in which stromal cells enhance E-cadherin expression and transdifferentiate into epithelial-like decidual cells [2,45]. During decidualization, laminins are highly expressed in human and mouse decidua [46,47,48]. Laminins bind laminin-specific receptors to maintain the integrity of epithelium [49]. Recent reports suggest that laminins regulate MET in Drosophila [50]. Therefore, laminin α5β2γ1 might be involved in MET.

Progesterone–progesterone receptor signaling is required for decidualization [35,51]. Progesterone can maintain the decidual response under artificial decidualization [52]. In our study, laminin A5 expression is stimulated by progesterone, which was impeded by PR antagonist RU486. Both mouse and human in vitro decidualization are suppressed by *Laminin A5* siRNA, suggesting that laminin A5 should be an important regulator during progesterone-induced decidualization.

The concentration of cAMP in the uterine lumen increases following artificially and blastocyst-induced decidualization [53,54]. In addition, cAMP can induce implantation of the embryos which are in the dormant state [55]. The intracellular cAMP concentration in uterine stromal cells is significantly increased by progesterone [37]. It is shown that cAMP is a strong inducer of human decidualization [36]. Our previous studies also showed that progesterone induced decidualization via cAMP-PKA signaling [38,39]. Previous studies have revealed that cAMP-PKA signaling is important for human decidualization [36,56]. In this study, H89 abolishes progesterone induction of laminin A5 expression. *Prl8a2,* a marker of mouse decidualization, is also inhibited by H89, suggesting that cAMP-PKA signaling is involved in regulating progesterone on laminin A5 expression.

CREB is a significant mediator for decidualization evoked by cAMP in the mouse uterus [57]. C/EBPβ is a transcription factor that has been reported as a crucial regulator of mouse decidualization in response to progesterone [58,59]. Ablation of C/EBPβ in female mice resulted in impaired decidualization and infertility [21]. A previous study showed that CREB controls C/EBPβ LAP transcription [60]. In macrophages, CREB mediated induction of C/EBPβ expression [40]. In this study, progesterone induction on laminin A5 is abolished by KG-501, C/EBPβ LIP, or C/EBPβ siRNA. C/EBPβ siRNA significantly inhibited the induction of progesterone on p-C/EBPβ expression. However, C/EBPβ siRNA did not affect progesterone up-regulation of p-CREB, suggesting that CREB-C/EBPβ signaling should mediate progesterone regulation of laminin A5 expression during mouse decidualization. Based on our in silico analysis, there is a potential C/EBPβ binding site in the laminin A5 promoter. C/EBPβ might bind to laminin A5 promoter and promote laminin A5 expression. A previous study also showed that laminin C1 is transcriptionally regulated by C/EBPβ during mouse decidualization [21].

**Figure 9:**
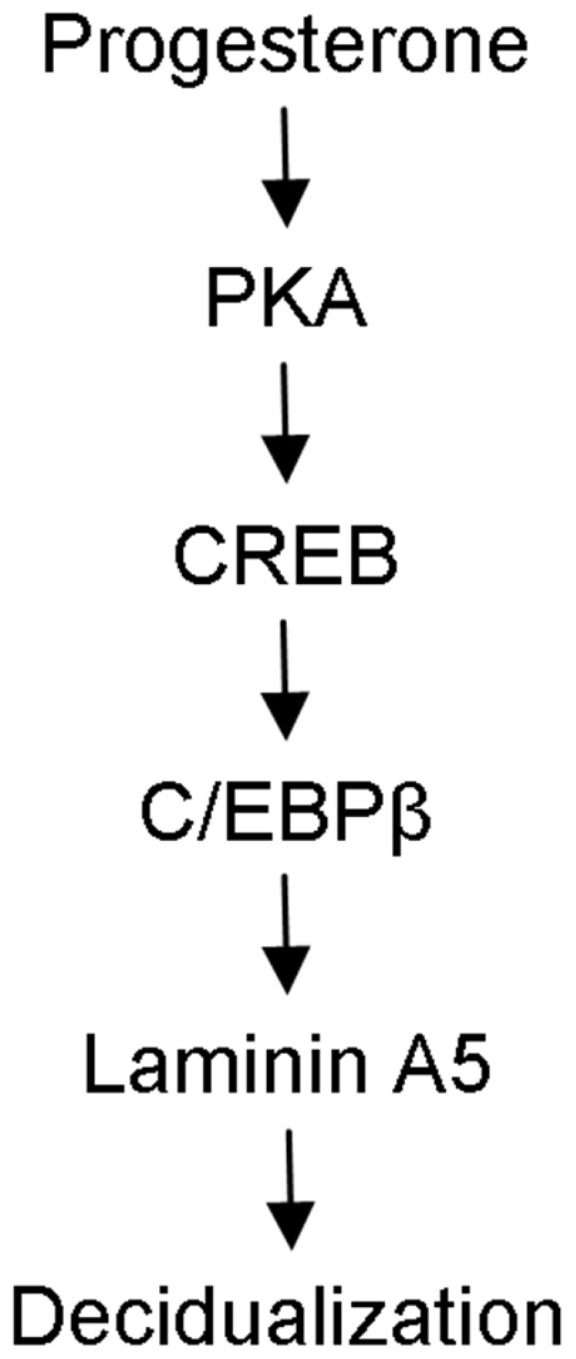
The schematic pathway showing the regulation of laminin A5 on mouse decidualization.

In conclusion, we showed that laminin A5 is highly expressed in decidua on days 5 to 8 of mouse pregnancy. Laminin A5 might act downstream of PKA-CREB-C/EBPβ to mediate the effects of progesterone on the decidualization of uterine stromal cells (Figure 9). Further deciphering the function of laminin A5 may provide the basis to improve fertility during pregnancy.

## 4. Materials and Methods

### 4.1. Animals

Sexually mature ICR mice were used in this study and housed in a light and temperature-controlled SPF environment (12 h light). All mouse protocols were approved by the Institutional Animal Care and Use Committee of South China Agricultural University. Female mice were bred overnight with fertile or vasectomized males to induce pregnancy or pseudopregnancy (vaginal plug = day 1). Implantation sites on days 5 and 6 of pregnancy were identified by tail vein injections of 100 μL of 1% Chicago Sky Blue 6B (Sigma-Aldrich, St. Louis, MO, USA).

To determine the effects of progesterone on laminin A5, p-CREB, and p-C/EBPβ, female mice were ovariectomized and rested for 14 days to remove ovarian steroid hormones. Then, ovariectomized mice were subcutaneously injected with progesterone (1 mg/100 μL/mouse in sesame oil). Control mice were injected with 100 μL sesame oil (Sigma-Aldrich). Artificial decidualization was induced by injecting 10 μL of sesame oil into one uterine horn on day 4 of pseudopregnancy. The uteri were collected on days 8.

### 4.2. Collection of Human Decidual Samples

Human decidual tissues were collected from women aged 31–38 years old who underwent elective terminations of early pregnancy (8–10 gestational weeks) in Women’s Hospital, School of Medicine Zhejiang University (Hangzhou, China) with informed consent. All procedures involving human decidual samples were approved by the Ethical Committee of Women’s Hospital, School of Medicine, Zhejiang University (File no. 20180192).

### 4.3. Isolation and Treatment of Mouse Endometrial Stromal Cells

Mouse endometrial stromal cells were isolated and treated as previously described [43]. In brief, mouse uteri on day 4 were split longitudinally and digested with 6 mg/mL dispase II (Roche Applied Science, Indianapolis, IN) and 1% trypsin (Amresco, Cleveland, OH) in HBSS (Sigma-Aldrich). After luminal epithelial cells were removed by rinsing, the remaining uteri were digested with 0.15 mg/mL collagenase I (Invitrogen, Houston, TX, USA). The collected stromal cells were cultured with DMEM/F12 (Sigma-Aldrich) containing 10% charcoal-treated FBS (cFBS; Biological Industries, Cromwell, Israel). To induce in vitro decidualization, mouse stromal cells were treated with 1 mM progesterone (Sigma-Aldrich) and 10 nM 17β-estradiol (Sigma-Aldrich) in DMEM/F12 (Sigma-Aldrich) containing 2% cFBS (Biological Industries).

### 4.4. Culture and Treatment of Human Endometrial Stromal Cells

The immortalized human endometrial stromal cells line (T HESCs, ATCC CRL-4003) was purchased from ATCC (Manassas, VA). Human stromal cells were cultured with DMEM/F12 (Sigma-Aldrich) containing 10% cFBS (Biological Industries). To induce in vitro decidualization, stromal cells were treated with 500 μM db-cAMP (dibutyryl cAMP sodium salt, Sigma-Aldrich) and 1 μM medroxyprogesterone acetate (MPA, Absin, Shanghai, China) as previously described [61].

### 4.5. siRNA Transfection

The siRNAs for mouse *Laminin A5*, *C/EBPβ*, and human *LAMININ A5* were designed and synthesized by Ribobio Co., Ltd. (Guangzhou, China). Cells were transfected with each siRNA according to the Lipofectamine 2000 instructions (Invitrogen). After being transfected for 6 h, the stromal cells were induced for in vitro decidualization. These siRNA sequences were listed in Table 1.

### 4.6. C/EBPβ LIP Overexpression

The overexpression plasmid of C/EBPβ LIP was obtained from Addgene (#12561, Watertown, MA, USA) and the pcDNA3.1 (+) vector was obtained from Invitrogen. Transfection of overexpression plasmid was performed according to the Lipofectamine 2000 instruction (Invitrogen).

### 4.7. Real-Time Quantitative Polymerase Chain Reaction (RT-qPCR)

RT-qPCR was performed as previously described [43]. Total RNAs were extracted using AG RNAex Pro Reagent (Accurate Biotechnology, Hunan, China) and reverse-transcribed into cDNA with HiScript II Q RT SuperMix for qPCR (Vazyme Biotech Co., Ltd., Nanjing, China). RT-qPCR was performed using ChamQ SYBR qPCR Master Mix (Vazyme Biotech Co., Ltd.) on the CFX96 Touch Real-Time PCR Detection System (Bio-Rad, Hercules, CA, USA). The primer sequences used for RT-qPCR are listed in Table 1. The fold change of gene expression was analyzed by 2^−^^ΔΔ^^CT^ method and normalized to *Rpl7*.

### 4.8. Immunofluorescence

Immunofluorescence was performed as previously described [62]. In brief, frozen uterine sections (10 μm) were cut and fixed in freshly prepared 4% paraformaldehyde in PBS for 30 min. After being permeabilized with 0.1% Triton X-100 in PBS for 15 min and blocked with 10% horse serum (Zhongshan Golden Bridge, Beijing, China) at 37 °C for 1 h, sections were incubated with primary antibody for anti-laminin A5 (ab184330, Abcam, MA, USA), anti-laminin B2 (05-206, Sigma-Aldrich), anti-laminin C1 (ab233398, Abcam), anti-CK18 (sc-6259, Santa Cruz Biotechnology, Dallas, TX, USA), or anti-IGFBP1 (sc-55474, Santa Cruz Biotechnology) overnight at 4 °C. After washing with PBS, sections were incubated with 488- or 594-conjugated second antibody (Jackson ImmunoResearch Laboratories, West Grove, PA, USA) at 37 °C for 30 min. The section was then counterstained with 4′,6-Diamidino-2-Phenylindole, Dihydrochloride (DAPI, Sigma-Aldrich), and mounted with an antifading mounting medium (Solarbio Life Sciences, Beijing, China).

### 4.9. Western Blot

Western blot was performed as previously described [63]. In brief, tissues or cultured cells were lysed in lysis buffer (50 mM Tris-HCl, pH 7.5; 150 mM NaCl; 0.25% sodium deoxycholate and 1% Triton X-100). The protein concentrations of lysate samples were measured by BCA kit (Thermo Fisher Scientific, Waltham, MA, USA). Protein samples were separated by 10% SDS/PAGE gels and transferred onto PVDF membranes (Merck KGaA, Darmstadt, Germany). After blocking with 5% nonfat milk (BBI Life Sciences, Shanghai, China) for 1 h at 25 °C, the membranes were incubated at 4 °C overnight with primary antibody for anti-laminin A5 (ab184330, Abcam), anti-P-C/EBPβ (3084S, Cell Signaling Technology, MA), anti-P-CREB (9198S, Cell Signaling Technology), anti-CREB (9197S, Cell Signaling Technology), anti-FoxO1 (2880S, Cell Signaling Technology), or anti-α-tubulin (2144S, Cell Signaling Technology). After washing, the membranes were incubated with the HRP-conjugated secondary antibody (1:5000, Invitrogen) for 1 h. The signals were detected with ECL chemiluminescent kit (Merck KGaA).

### 4.10. Statistical Analysis

All experiments were repeated at least three times independently. The data were presented as the mean ± standard deviation (SD). The differences between the two groups were compared by Student’s t-test. One-way ANOVA test followed by Newman–Keuls test was performed for multiple comparisons. *p* < 0.05 was considered to be statistically significant.

## Figures and Tables

**Table 1:** Primers and siRNA sequences used in this study.

Gene	Species	Sequence (5’-3’)	Application	Accession Number	Product Size
*Rpl7*	Mouse	GCAGATGTACCGCACTGAGATTC ACCTTTGGGCTTACTCCATTGATA	RT-qPCR	NM_011291.5	129 bp
*Prl8a2*	Mouse	AGCCAGAAATCACTGCCACT TGATCCATGCACCCATAAAA	RT-qPCR	NM_010088	119 bp
*Laminin A5*	Mouse	AGCAAGGCGATCCAAGTGTT TTGAGGTCCACGTACTTGCC	RT-qPCR	NM_001081171.2	242 bp
*Laminin B1*	Mouse	GCGCTGAACAATAGCTGCTC AATAAGCCCCTTCAGGCACC	RT-qPCR	NM_008482.3	241 bp
*Laminin B2*	Mouse	CTGGTGACCAACCGAGAGAC CAGCGCAGTAGCAGGTCATA	RT-qPCR	NM_008483.3	128 bp
*Laminin C1*	Mouse	CGGAGTTTGTTAATGCCGCC TCGGCCTGGTTGTTGTAGTC	RT-qPCR	NM_010683.2	187 bp
*C/EBPβ*	Mouse	GACAAGCTGAGCGACGAGTA TGCTTGAACAAGTTCCGCAG	RT-qPCR	NM_001287738.1	197 bp
*IGFBP1*	Human	CCAAACTGCAACAAGAATG GTAGACGCACCAGCAGAG	RT-qPCR	NM_001013029	87 bp
*RPL7*	Human	CTGCTGTGCCAGAAACCCTT TCTTGCCATCCTCGCCAT	RT-qPCR	NM_000971	194 bp
*LAMININ A5*	Human	AGTGCCAGTCCTGTAACTGC GATAGGTGCCATCCAGGCTC	RT-qPCR	NM_005560.6	99 bp
*NC*	-	CTCCGAACGTGTCACGT	siRNA		
*Laminin A5*	Mouse	GCGACTGTGAGTCAGACTT	siRNA		
*C/EBPβ*	Mouse	GAGCGACGAGUACAAGAUG	siRNA		
*LAMININ A5*	Human	GCCTCGTGCTGTTGTATGA	siRNA		

## Data Availability

All the data generated in this study are included in this manuscript.

## Supplementary Materials

The following supporting information can be downloaded at: https://www.mdpi.com/article/10.3390/ijms23010199/s1.

## Abbreviations

C/EBPβ	CCAAT enhancer-binding protein β
CREB	cAMP-response element-binding protein
H89	PKA inhibitor
IGFBP1	Insulin growth factor-binding protein 1
KG-501	CREB inhibitor
PKA	Protein kinase A
Prl8a2	Prolactin family 8, subfamily A, member 2
RU486	progesterone receptor antagonist

## References

[1] Wang H., Dey S.K. (2006). Roadmap to embryo implantation: Clues from mouse models. Nat. Rev. Genet..

[2] Owusu-Akyaw A., Krishnamoorthy K., Goldsmith L.T., Morelli S.S. (2019). The role of mesenchymal-epithelial transition in endometrial function. Hum. Reprod. Update.

[3] Pawar S., Hantak A.M., Bagchi I.C., Bagchi M.K. (2014). Minireview: Steroid-regulated paracrine mechanisms controlling implantation. Mol. Endocrinol..

[4] Okada H., Tsuzuki T., Murata H. (2018). Decidualization of the human endometrium. Reprod. Med. Biol..

[5] Sekiguchi R., Yamada K.M. (2018). Basement membranes in development and disease. Curr. Top Dev. Biol..

[6] Jayadev R., Sherwood D.R. (2017). Basement membranes. Curr. Biol..

[7] Theocharis A.D., Skandalis S.S., Gialeli C., Karamanos N.K. (2016). Extracellular matrix structure. Adv. Drug Deliv. Rev..

[8] Schittny J.C., Yurchenco P.D. (1989). Basement membranes: Molecular organization and function in development and disease. Curr. Opin. Cell Biol..

[9] Paulsson M. (1992). Basement membrane proteins: Structure, assembly, and cellular interactions. Crit. Rev. Biochem. Mol. Biol..

[10] Timpl R., Rohde H., Robey P.G., Rennard S.I., Foidart J.M., Martin G.R. (1979). Laminin—A glycoprotein from basement membranes. J. Biol. Chem..

[11] Chung A.E., Jaffe R., Freeman I.L., Vergnes J.P., Braginski J.E., Carlin B. (1979). Properties of a basement membrane-related glycoprotein synthesized in culture by a mouse embryonal carcinoma-derived cell line. Cell.

[12] Timpl R., Dziadek M., Fujiwara S., Nowack H., Wick G. (1983). Nidogen: A new, self-aggregating basement membrane protein. Eur. J. Biochem..

[13] Hassell J.R., Robey P.G., Barrach H.J., Wilczek J., Rennard S.I., Martin G.R. (1980). Isolation of a heparan sulfate-containing proteoglycan from basement membrane. Proc. Natl. Acad. Sci. USA.

[14] Mason I.J., Taylor A., Williams J.G., Sage H., Hogan B.L. (1986). Evidence from molecular cloning that SPARC, a major product of mouse embryo parietal endoderm, is related to an endothelial cell ‘culture shock’ glycoprotein of Mr 43,000. EMBO J..

[15] Tsen G., Halfter W., Kröger S., Cole G.J. (1995). Agrin is a heparan sulfate proteoglycan. J. Biol. Chem..

[16] Halfter W., Dong S., Schurer B., Cole G.J. (1998). Collagen XVIII is a basement membrane heparan sulfate proteoglycan. J. Biol. Chem..

[17] Timpl R., Sasaki T., Kostka G., Chu M.L. (2003). Fibulins: A versatile family of extracellular matrix proteins. Nat. Rev. Mol. Cell Biol..

[18] Jones-Paris C.R., Paria S., Berg T., Saus J., Bhave G., Paria B.C., Hudson B.G. (2017). Embryo implantation triggers dynamic spatiotemporal expression of the basement membrane toolkit during uterine reprogramming. Matrix Biol..

[19] Yin Y., Wang A., Feng L., Wang Y., Zhang H., Zhang I., Bany B.M., Ma L. (2018). Heparan sulfate proteoglycan sulfation regulates uterine differentiation and signaling during embryo implantation. Endocrinology.

[20] Nakamoto T., Okada H., Nakajima T., Ikuta A., Yasuda K., Kanzaki H. (2005). Progesterone induces the fibulin-1 expression in human endometrial stromal cells. Hum. Reprod..

[21] Ramathal C., Wang W., Hunt E., Bagchi I.C., Bagchi M.K. (2011). Transcription factor CCAAT enhancer-binding protein beta (C/EBPbeta) regulates the formation of a unique extracellular matrix that controls uterine stromal differentiation and embryo implantation. J. Biol. Chem..

[22] Shi J.W., Lai Z.Z., Yang H.L., Yang S.L., Wang C.J., Ao D., Ruan L.Y., Shen H.H., Zhou W.J., Mei J. (2020). Collagen at the maternal-fetal interface in human pregnancy. Int. J. Biol. Sci..

[23] Burgeson R.E., Chiquet M., Deutzmann R., Ekblom P., Engel J., Kleinman H., Martin G.R., Meneguzzi G., Paulsson M., Sanes J. (1994). A new nomenclature for the laminins. Matrix Biol..

[24] Yao Y. (2017). Laminin: Loss-of-function studies. Cell Mol. Life Sci..

[25] Hohenester E. (2019). Structural biology of laminins. Essays Biochem..

[26] Yap L., Tay H.G., Nguyen M., Tjin M.S., Tryggvason K. (2019). Laminins in cellular differentiation. Trends Cell. Biol..

[27] Aumailley M. (2013). The laminin family. Cell. Adh. Migr..

[28] Miner J.H., Patton B.L., Lentz S.I., Gilbert D.J., Snider W.D., Jenkins N.A., Copeland N.G., Sanes J.R. (1997). The laminin alpha chains: Expression, developmental transitions, and chromosomal locations of alpha1-5, identification of heterotrimeric laminins 8–11, and cloning of a novel alpha3 isoform. J. Cell. Biol..

[29] Miner J.H., Cunningham J., Sanes J.R. (1998). Roles for laminin in embryogenesis: Exencephaly, syndactyly, and placentopathy in mice lacking the laminin alpha5 chain. J. Cell. Biol..

[30] Noakes P.G., Gautam M., Mudd J., Sanes J.R., Merlie J.P. (1995). Aberrant differentiation of neuromuscular junctions in mice lacking s-laminin/laminin beta 2. Nature.

[31] Smyth N., Vatansever H.S., Murray P., Meyer M., Frie C., Paulsson M., Edgar D. (1999). Absence of basement membranes after targeting the LAMC1 gene results in embryonic lethality due to failure of endoderm differentiation. J. Cell. Biol..

[32] Murray P., Edgar D. (2000). Regulation of programmed cell death by basement membranes in embryonic development. J. Cell. Biol..

[33] Gu Y., Soares M.J., Srivastava R.K., Gibori G. (1994). Expression of decidual prolactin-related protein in the rat decidua. Endocrinology.

[34] Dey S.K., Lim H., Das S.K., Reese J., Paria B.C., Daikoku T., Wang H. (2004). Molecular cues to implantation. Endocr. Rev..

[35] Zhang S., Lin H., Kong S., Wang S., Wang H., Wang H., Armant D.R. (2013). Physiological and molecular determinants of embryo implantation. Mol. Aspects Med..

[36] Gellersen B., Brosens J. (2003). Cyclic AMP and progesterone receptor cross-talk in human endometrium: A decidualizing affair. J. Endocrinol..

[37] Yu H.F., Tao R., Yang Z.Q., Wang K., Yue Z.P., Guo B. (2018). Ptn functions downstream of C/EBPβ to mediate the effects of cAMP on uterine stromal cell differentiation through targeting Hand2 in response to progesterone. J. Cell. Physiol..

[38] Deng W.B., Liang X.H., Liu J.L., Yang Z.M. (2014). Regulation and function of deiodinases during decidualization in female mice. Endocrinology.

[39] Huang Z., Wang T.S., Zhao Y.C., Zuo R.J., Deng W.B., Chi Y.J., Yang Z.M. (2014). Cyclic adenosine monophosphate-induced argininosuccinate synthase 1 expression is essential during mouse decidualization. Mol. Cell. Endocrinol..

[40] Ruffell D., Mourkioti F., Gambardella A., Kirstetter P., Lopez R.G., Rosenthal N., Nerlov C. (2009). A CREB-C/EBPbeta cascade induces M2 macrophage-specific gene expression and promotes muscle injury repair. Proc. Natl. Acad. Sci. USA.

[41] Al-Sabbagh M., Fusi L., Higham J., Lee Y., Lei K., Hanyaloglu A.C., Lam E.W., Christian M., Brosens J.J. (2011). NADPH oxidase-derived reactive oxygen species mediate decidualization of human endometrial stromal cells in response to cyclic AMP signaling. Endocrinology.

[42] Gellersen B., Brosens J.J. (2014). Cyclic decidualization of the human endometrium in reproductive health and failure. Endocr. Rev..

[43] Gu X.W., Chen Z.C., Yang Z.S., Yang Y., Yan Y.P., Liu Y.F., Pan J.M., Su R.W., Yang Z.M. (2020). Blastocyst-induced ATP release from luminal epithelial cells initiates decidualization through the P2Y2 receptor in mice. Sci. Signal.

[44] Qin Y., Shembrey C., Smith J., Paquet-Fifield S., Behrenbruch C., Beyit L.M., Thomson B., Heriot A.G., Cao Y., Hollande F. (2020). Laminin 521 enhances self-renewal via STAT3 activation and promotes tumor progression in colorectal cancer. Cancer Lett..

[45] Zhang X.H., Liang X., Liang X.H., Wang T.S., Qi Q.R., Deng W.B., Sha A.G., Yang Z.M. (2013). The mesenchymal-epithelial transition during in vitro decidualization. Reprod. Sci..

[46] Kisalus L.L., Herr J.C., Little C.D. (1987). Immunolocalization of extracellular matrix proteins and collagen synthesis in first-trimester human decidua. Anat. Rec..

[47] Church H.J., Vićovac L.M., Williams J.D., Hey N.A., Aplin J.D. (1996). Laminins 2 and 4 are expressed by human decidual cells. Lab. Investig..

[48] Blankenship T.N., Given R.L. (1995). Loss of laminin and type IV collagen in uterine luminal epithelial basement membranes during blastocyst implantation in the mouse. Anat. Rec..

[49] Scott L.E., Weinberg S.H., Lemmon C.A. (2019). Mechanochemical signaling of the extracellular matrix in epithelial-mesenchymal transition. Front. Cell. Dev. Biol..

[50] Pitsidianaki I., Morgan J., Adams J., Campbell K. (2021). Mesenchymal-to-epithelial transitions require tissue-specific interactions with distinct laminins. J. Cell. Biol..

[51] Large M.J., DeMayo F.J. (2012). The regulation of embryo implantation and endometrial decidualization by progesterone receptor signaling. Mol. Cell. Endocrinol..

[52] Paria B.C., Tan J., Lubahn D.B., Dey S.K., Das S.K. (1999). Uterine decidual response occurs in estrogen receptor-alpha-deficient mice. Endocrinology.

[53] Vilar-Rojas C., Castro-Osuna G., Hicks J.J. (1982). Cyclic AMP and cyclic GMP in the implantation site of the rat. Int. J. Fertil..

[54] Rankin J.C., Ledford B.E., Baggett B. (1977). Early involvement of cyclic nucleotides in the artificially stimulated decidual cell reaction in the mouse uterus. Biol. Reprod..

[55] Holmes P.V., Bergström S. (1975). Induction of blastocyst implantation in mice by cyclic AMP. J. Reprod. Fertil..

[56] Telgmann R., Maronde E., Taskén K., Gellersen B. (1997). Activated protein kinase A is required for differentiation-dependent transcription of the decidual prolactin gene in human endometrial stromal cells. Endocrinology.

[57] Ruan Y.C., Guo J.H., Liu X., Zhang R., Tsang L.L., Dong J.D., Chen H., Yu M.K., Jiang X., Zhang X.H. (2012). Activation of the epithelial Na+ channel triggers prostaglandin E_2_ release and production required for embryo implantation. Nat. Med..

[58] Mantena S.R., Kannan A., Cheon Y.P., Li Q., Johnson P.F., Bagchi I.C., Bagchi M.K. (2006). C/EBPbeta is a critical mediator of steroid hormone-regulated cell proliferation and differentiation in the uterine epithelium and stroma. Proc. Natl. Acad. Sci. USA.

[59] Li D.D., Yang Z.Q., Guo C.H., Yue L., Duan C.C., Cao H., Guo B., Yue Z.P. (2015). Hmgn1 acts downstream of C/EBPβ to regulate the decidualization of uterine stromal cells in mice. Cell. Cycle.

[60] Niehof M., Manns M.P., Trautwein C. (1997). CREB controls LAP/C/EBP beta transcription. Mol. Cell. Biol..

[61] Qi Q.R., Zhao X.Y., Zuo R.J., Wang T.S., Gu X.W., Liu J.L., Yang Z.M. (2015). Involvement of atypical transcription factor E2F8 in the polyploidization during mouse and human decidualization. Cell. Cycle.

[62] Zheng H.T., Zhang H.Y., Chen S.T., Li M.Y., Fu T., Yang Z.M. (2020). The detrimental effects of stress-induced glucocorticoid exposure on mouse uterine receptivity and decidualization. FASEB J..

[63] Liang Y.X., Hu W., Jin Z.Y., Diao H.L., Liu L., Yang Y., Fu T., Yang Z.M. (2020). Nucleolar stress regulates stromal-epithelial transition via NPM1 during decidualization. Reproduction.

