# Supplementary material for: Regulation and Function of Laminin A5 during Mouse and Human Decidualization
Source: Int J Mol Sci. 2021 Dec 24;23(1):199. doi: 10.3390/ijms23010199 (PMC8745792; doi:10.3390/ijms23010199)
Supplement: Supplementary file 1 [file ijms-23-00199-s001.zip › Figure S1.pdf]

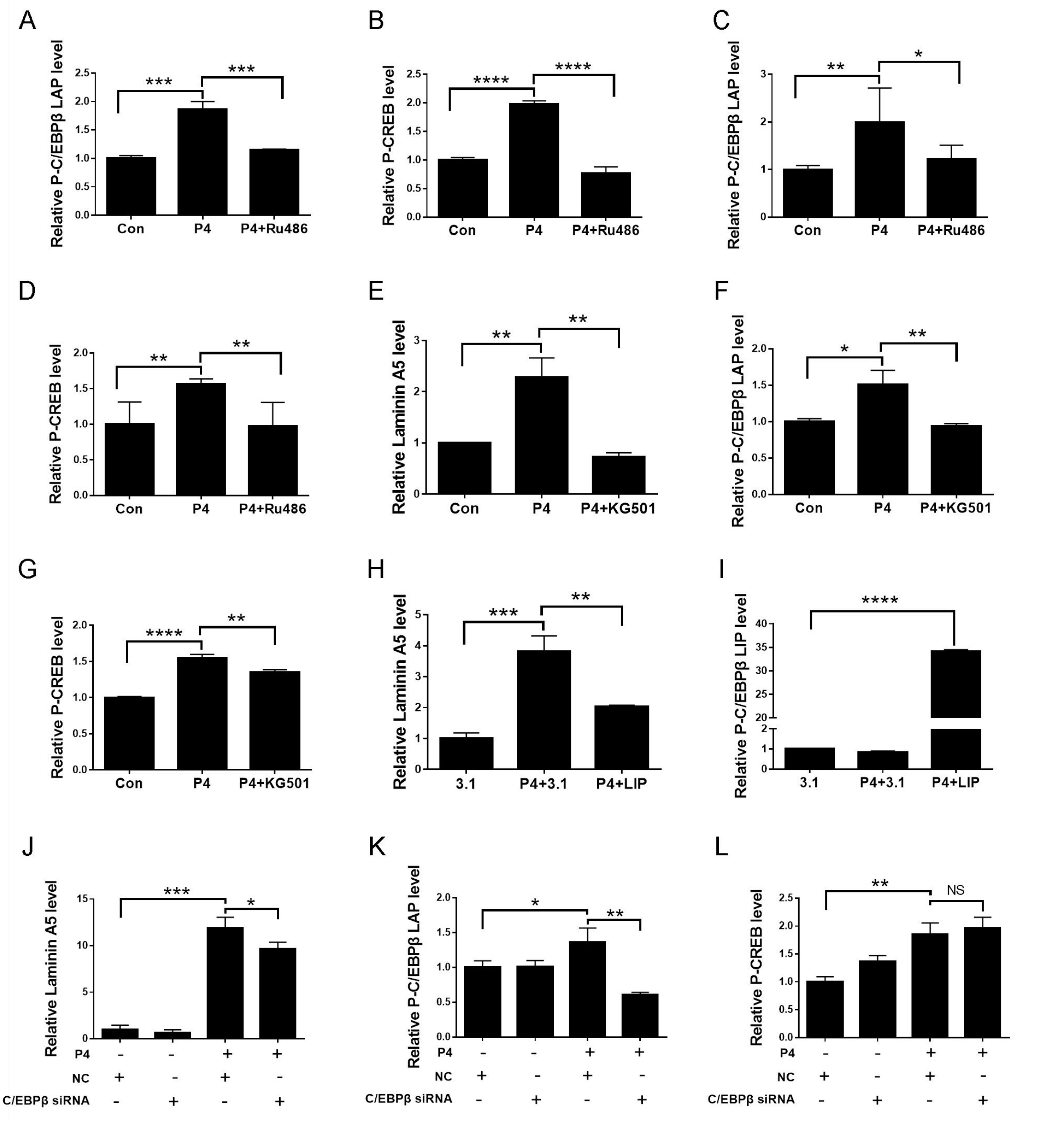


**Figure S1.** Quantification of p-C/EBPβ LAP, p-CREB, Laminin A5, and p-C/EBPβ LIP. Bars represent mean ± SD (*p-value < 0.05, **p-value < 0.01, ***p-value < 0.001, ****p-value < 0.0001). NS, Not Significant.
